# Non-Nutritive Sweeteners Acesulfame Potassium and Sucralose Are Competitive Inhibitors of the Human P-glycoprotein/Multidrug Resistance Protein 1 (PGP/MDR1)

**DOI:** 10.3390/nu15051118

**Published:** 2023-02-23

**Authors:** Laura Danner, Florian Malard, Raquel Valdes, Stephanie Olivier-Van Stichelen

**Affiliations:** 1 Department of Biochemistry, Medical College of Wisconsin, Milwaukee, WI 53226, USA; 2 INSERM U1212, CNRS UMR5320, ARNA Laboratory, University of Bordeaux, 33000 Bordeaux, France; 3 Department of Obstetrics & Gynecology, Medical College of Wisconsin, Milwaukee, WI 53226, USA

**Keywords:** non-nutritive sweeteners, artificial sweeteners, acesulfame potassium, sucralose, P-glycoprotein, ABCB1, MDR1

## Abstract

Non-nutritive sweeteners (NNS) are popular sugar replacements used in foods, beverages, and medications. Although NNS are considered safe by regulatory organizations, their effects on physiological processes such as detoxification are incompletely understood. Previous studies revealed that the NNS sucralose (Sucr) altered P-glycoprotein (PGP) expression in rat colon. We also demonstrated that early-life exposure to NNS Sucr and acesulfame potassium (AceK) compromises mouse liver detoxification. Building upon these initial discoveries, we investigated the impact of AceK and Sucr on the PGP transporter in human cells to assess whether NNS influence its key role in cellular detoxification and drug metabolism. We showed that AceK and Sucr acted as PGP inhibitors, competing for the natural substrate-binding pocket of PGP. Most importantly, this was observed after exposure to concentrations of NNS within expected levels from common foods and beverage consumption. This may suggest risks for NNS consumers, either when taking medications that require PGP as the primary detoxification transporter or during exposure to toxic compounds.

## 1. Introduction

For decades, non-nutritive sweeteners (NNS) have been commonly used as food additives in the United States. They are found ubiquitously in products including “diet” or “zero sugar” foods and beverages, medicines, lip balms, chewing gums, tabletop sweeteners, and yogurts. NNS are small molecules with a significantly greater sweetness intensity than traditional nutritive sweeteners such as sucrose and fructose and do not contribute to caloric intake [1]. Six NNS are currently approved for consumption by the Food and Drug Administration (FDA): aspartame, sucralose (Sucr), acesulfame potassium (AceK), saccharin, neotame, and advantame [2]. Following toxicology studies in primarily animal models, the FDA defined an Acceptable Daily Intake (ADI) for each NNS, indicating daily amounts considered safe for human consumption.

Consumption of NNS is highly prevalent among people in developed countries [3]. Analyses of dietary trends show that, in the United States, 25% of children and over 40% of adults consume NNS [3]. Rates of NNS consumption are usually higher among females, people with obesity, and people with a diagnosis of diabetes [3]. Additionally, the majority of adults consuming NNS report doing so on a daily basis [3]. Furthermore, the proportion of US households purchasing foods and beverages containing NNS increased significantly from 2008 to 2018, mainly driven by an increase in beverages containing a combination of NNS and caloric sweeteners [4]. Interestingly, involuntary environmental exposure to NNS is also likely, as trace levels of NNS have been found in treated and untreated wastewater [5]. Indeed, NNS have been detected in blood, feces, and breastmilk samples from control groups in murine studies [6].

While nutritive sweeteners such as sucrose are easily metabolized for energy when consumed, most NNS are highly stable, not metabolized, and excreted intact. Two such NNS, sucralose (Sucr) and acesulfame potassium (AceK), are frequently combined in diet foods and beverages but show different distribution patterns in human tissues. Indeed, AceK is rapidly and nearly completely absorbed into the circulation, and, as it undergoes no metabolism, remains in its original structure as it bathes various organs [1]. Ultimately, 98% of consumed AceK is excreted by the kidneys and removed through the urine. On the contrary, Sucr is absorbed at a lower rate, with only 10–20% of orally administered Sucr recovered in the urine of human subjects [7]. In one study, roughly 2% of Sucr recovered in urine was found as glucuronide conjugates, demonstrating low metabolism rates in the liver. The majority of Sucr is excreted unmetabolized in feces. On average, over 90% of orally administered Sucr is passed within five days of consumption.

While advertised in weight-loss programs [8], the effectiveness of NNS for successful weight loss and their impact on blood sugar control has been controversial, casting doubts over their potential for benefits beyond reducing dental caries [9,10,11]. NNS have been associated with an increased prevalence of non-alcoholic fatty liver disease (NAFLD), the hepatic manifestation of metabolic syndromes, and diabetes [12]. NNS also alter murine gut hormonal secretion and absorption [13] and promote “obesogenic” microbiome dysbiosis [14]. Importantly, adverse effects of NNS combinations or the use of NNS in specific populations were identified sometimes decades after FDA approval and were not revealed in the original toxicology studies, stressing the need for additional research on NNS safety [12,14]. Our lab recently highlighted a potentially damaging effect of NNS on detoxification pathways, with evidence of dysregulated liver function [6].

The liver is the main site of detoxification for drugs or xenobiotics that enter the circulation, utilizing conjugating enzymes and efflux transporters to neutralize and excrete toxins [15]. Liver metabolites can be either excreted through bile in feces or returned to general circulation and filtered through the kidneys. One essential detoxifying efflux transporter, P-glycoprotein (PGP), is impacted by the consumption of NNS [16]. PGP, also known as multidrug resistance protein 1 (MDR1) or ATP-binding cassette sub-family B member 1 (ABCB1), is an ATP-dependent 170 kDa transmembrane protein that expels a variety of intracellular compounds from tissues for excretion. PGP is also a major determinant of the absorption, disposition, and elimination of many common drugs in key tissues such as the intestines and blood–brain barrier [17]. In the liver, PGP effluxes drugs, toxins, biliary pigments, and metabolic conjugates from hepatocytes into the biliary ducts [18]. Inhibition of PGP by different drugs can lead to a compensatory increase in *ABCB1*/PGP expression as the tissue attempts to maintain its excretory functioning [19].

Previous work in our lab found prominent effects of NNS-induced toxicity in the livers of offspring from mothers fed a diet with AceK and Sucr [6]. Therefore, we wondered whether NNS consumption alters PGP efflux efficiency, leading to reduced detoxification capacity in the liver. In the present work, we confirmed that combined AceK and Sucr increase *ABCB1*/PGP levels in a human liver cell line, similar to expected expression levels following exposure to PGP inhibitors [19]. Further, we demonstrated that AceK and Sucr stimulated PGP ATPase activity while inhibiting the efflux of known PGP substrates, thus acting as competitive inhibitors of PGP. These effects were found at concentrations of NNS that have been recorded in human tissue samples after consumption of a single diet beverage [20,21,22]. Finally, AceK and Sucr were found to occupy PGP binding pockets in silico, making similar biochemical contacts as the known competitive inhibitor drugs Verapamil and Vincristine. In conclusion, our findings suggest that consuming AceK and Sucr within recommended levels poses potential risks for those also taking medications transported by PGP. Further research may be necessary to determine safe levels of NNS consumption for people taking these medications.

## 2. Materials and Methods

### 2.1. Cell Culture

HepG2 and HEK-293 cells (ATCC, Manassas, VA, USA) were cultured in Dulbecco’s Modified Eagle Medium (DMEM) and supplemented with 10% FBS (Gibco, Waltham, MA, USA), 1% penicillin/streptomycin (Gibco, Waltham, MA, USA), and 200 mM L-glutamine (Gibco, Waltham, MA, USA). Cells were maintained in a humidified incubator with 5% CO_2_ at 37 °C.

### 2.2. NNS and Other Reagents

Acesulfame potassium (AceK) and sucralose (Sucr) were kindly provided by Kristina L. Rother (NICHD, National Institutes of Health, Bethesda, MD, USA) or alternatively bought from Sigma-Aldrich (St. Louis, MO, USA—Sucr) or Cayman Chemical Company (Ann Arbor, MI, USA—AceK). Experimental NNS concentrations were determined based on previously reported levels in tissue after dietary exposure for each NNS and can be found in Table 1. For in vitro PGP functional assays, Verapamil HCl was purchased from BioVision, Inc (Milpitas, CA, USA). and Calcein-AM was purchased from Cayman Chemical Company (Ann Arbor, MI, USA). Alternatively, Calcein-AM retention was measured with the Multidrug Efflux Transporter P Glycoprotein (MDR1/P-gp) Ligand Screening Kit (Abcam, Cambridge, UK, #ab284553). For cell-free PGP activity assays, the ADP-Glo MAX assay (Promega, Madison, WI, USA, #V7001) was used for ATPase stimulation. Sodium orthovanadate was also purchased from Promega (Madison, WI, USA).

### 2.3. RNA Extraction, cDNA Synthesis, and RT-qPCR

RNA was extracted with the PureLink RNA Mini Kit with on-column DNAse digestion (Invitrogen, Waltham, MA, USA) according to the manufacturer’s protocol. cDNA was synthesized using qScript cDNA SuperMix (QuantaBio, Beverly, MA, USA). Quantitative PCR (qPCR) was performed using PerfeCTa SYBR Green FastMix, Low ROX (QuantaBio, Beverly, MA, USA) on the QuantStudio3 qPCR systems (Applied Biosystems, Waltham, MA, USA) according to the manufacturer’s protocol. Data were analyzed using the 2^−ΔΔCt^ methods [23]. Primers used in this study can be found in Table S1**.**

### 2.4. Protein Extraction, SDS Page, and Western Blot

Cells were lysed at 4 °C in Octyl Glucoside buffer (25 mM Tris-HCl pH 7.4, 20 mM NaCl, 60 mM octyl glucoside). Lysates were sonicated and centrifuged at 18,000× *g* for 20 min at 4 °C. Laemmli buffer (4×) (200 mM Trist-HCl pH 6.8, 277 mM SDS, 5.4 M glycerol, 20% [v/v] beta-mercaptoethanol, 3 mM bromophenol blue) was added to the supernatant of each lysate for a final concentration of 1× and boiled at 95 °C for five minutes before loading on a 6% SDS-PAGE pre-cast gel (Invitrogen, Waltham, MA, USA) for electrophoretic separation of proteins. Then, proteins were transferred onto a 0.45 mm nitrocellulose membrane using 14 h wet transfer (10 V, 0.7 mA) at 4 °C. For the protein loading control, membranes were first washed two times with nanopure water for 2 min, then stained with Total Protein Stain (Invitrogen, Waltham, MA, USA) for 10 min and rinsed in nanopure water an additional three times for 2 min. Membranes were then blocked in freshly prepared non-fat dry milk (10% [*w/v*] in TBS-T—25 mM Tris pH 7.5, 150 mM NaCl, 0.05% *w/v* Tween-20) for 45 min. Membranes were incubated with mouse monoclonal anti-P-glycoprotein antibodies (Sigma-Aldrich, St. Louis, MO, USA, #p7965) at 1:1000 dilution in 10% Milk/TBS-T for one hour at room temperature (RT, 25 °C). After 3 TBS-T washes, membranes were incubated with a goat anti-mouse fluorophore-conjugated secondary antibody (LI-COR, Lincoln, NE, USA) at 1:10,000 dilution in TBS-T for one hour at RT. After 3 washes with TBS-T and one rinse of 5 min with PBS, membranes were imaged on the Odyssey FC Imager (LI-COR, Lincoln, NE, USA). Images of full Western blots can be found in Figure S1.

### 2.5. Calcein-AM Retention Assay

Inhibition of PGP substrate efflux was assessed by Calcein-AM retention using either individually purchased reagents or the Multidrug Efflux Transporter P Glycoprotein (MDR1/P-gp) Ligand Screening Kit (Abcam, Cambridge, UK), which contained identical reagents in proprietary concentrations. Briefly, HepG2 or HEK-293 cells were seeded at a confluence of 8 × 10^4^ cells per well in a black-walled clear-bottomed 96-well plate and grown to roughly 90% confluence. Reagents were prepared in assay buffer (Hanks Balanced Salt Solution (HBSS) supplemented with 20 mM HEPES (from 1M HEPES buffer solution, Gibco, Waltham, MA, USA)) immediately before assay. Cells were washed once with warmed (37 °C) HBSS. Then, the plate was incubated at 37 °C for 15 min with assay buffer containing either diluted NNS, the PGP competitive inhibitor Verapamil (50 μM) (positive control), or equal volume of vehicle (HBSS) (negative control). Reporter substrate Calcein-AM was then added to wells to a final concentration of 0.25 μM, protected from light, and incubated at 37 °C for 30 min. Fluorescence was then measured on the FLUOstar Omega microplate reader (BMG LabTech, Ortenberg, Germany) with excitation/emission spectra at 488/532 nm. For assay optimization, HepG2 or HEK293 were incubated with increasing concentrations of Calcein-AM, and Calcein retention was measured as fluorescent signal at baseline and every 10 min thereafter for 70 min (Figure S2). HepG2 or HEK293 were then incubated with combined AceK and Sucr, Verapamil, or vehicle control and a select range of Calcein-AM concentrations for 30 min to determine Calcein-AM sensitivity to pharmacological inhibition (Figure S3).

### 2.6. PGP ATPase Activation Assay

P-glycoprotein ATPase activity was assessed using the ADP-Glo Max Assay (Promega, Madison, WI, USA). 5× assay buffer (250 mM Tris-MES pH 6.8, 50 mM MgCl_2_, 10 mM EGTA, 250 mM KCl, 25 mM sodium azide, 10 mM DTT) was prepared ahead of time and diluted to a final concentration of 1× with nanopure water immediately before each assay. Briefly, PGP membranes, substrates, inhibitor, NNS stock, and reagents were thawed at room temperature (RT, 25 °C) and prepared immediately prior to assay. PGP substrates, NNS, or inhibitors in buffer were added to opaque white 96-well plates. Verapamil (50 μM) is efficiently transported by the PGP and used as a positive control for maximum PGP stimulation. Sodium orthovanadate (100 μM) completely inhibits PGP ATPase inhibition and was used as negative control of background PGP activity. Human MDR1 vesicles (Invitrogen, Waltham, MA, USA) (0.01 mg in 1× assay buffer) were added to the wells and incubated at 37 °C for 5 min. Then, 10 μL of 5 mM Mg-ATP was added to each well as substrates for PGP (final concentration 5 mM Mg-ATP), and incubated at 37 °C for an additional 40 min. After 10 min equilibration to RT, 25 μL of ADP-GLO reagent was added to each well and incubated at RT for 40 min. Then, 50 μL of ATP Detection Reagent was added to each well in a dark room. The plate was briefly spun to mix, covered, and incubated at RT for 1 hr. Luminescence was measured on the FLUOstar Omega microplate reader (BMG LabTech, Ortenberg, Germany). ATPase stimulation from the PGP competitive inhibitor Verapamil was verified prior to treatment with AceK and Sucr (Figure S4).

### 2.7. Molecular Docking

The Cryo-EM structure of the nanodisc reconstituted, drug-free human PGP/ABCB1 was retrieved from the Protein Data Bank (PDB #7A65) [24]. Hydrogen atoms and gasteiger charges were added using the prepare_receptor script from ADFR suite [25] following the AutoDock Vina Documentation (Release 1.2.0) [26]. The reference 3D models of acesulfame, sucralose, and verapamil were retrieved from the ZINC database [27] in mol2 format and processed with the prepare_ligand script from ADFR suite. The resulting pdbqt files for the receptor and ligands were used in docking experiments with AutoDock Vina [26,28]. We used the Orientations of Proteins in Membranes (OPM) database [29] (model 7a65.pdb) to adjust the docking box coordinates and dimensions in the transmembrane region of the receptor. Vina 1.2 was run with an exhaustiveness parameter of 32, and 20 binding poses were collected for each experiment. For each ligand, the corresponding binding poses on the receptor were inspected and checked for consistency against experimental data [30,31,32]. Then, the best binding poses with respect to the Vina built-in scoring function were selected for presentation in this manuscript. PGP and docked ligands were visualized with PyMOL [33].

### 2.8. Statistical Analysis

Data were analyzed with GraphPad Prism software version 9.5.0. Values are presented as mean ± SEM. Ordinary one-way ANOVA were performed with uncorrected Fisher’s LSD with a single pooled variance. Multiple unpaired t-tests were performed with two-stage linear step-up procedure of Benjamini, Kieger, and Yekutieli. Significance is presented as follows: * *p* < 0.05; ** *p* < 0.01; *** *p* < 0.001; **** *p* < 0.0001. EC50 values were determined by Nonlinear fit of dose-response stimulation (Equation: [Agonist] vs. response). The model for the equation is given as: Y = Bottom + X × (Top-Bottom)/(EC50 + X).

## 3. Results

### 3.1. Acesulfame Potassium and Sucralose Impact the Expression of Detoxification Actors in Human Liver Cell Line

Our lab previously demonstrated that early-life chronic exposure to combined AceK and Sucr led to whitening in the livers of mouse pups and altered expression of phase II detoxification enzymes in the liver [6]. Furthermore, metabolic analysis confirmed the accumulation of intermediary metabolites not processed by phase II detoxification enzymes, leading to increased oxidative stress [6]. These findings suggested that NNS-fed pregnant mice gave birth to offspring with increased toxicity from inefficient liver detoxification. While this was the first indication that NNS might directly impact liver detoxification processes, the effects observed on the offspring’s liver might have been secondary consequences of NNS exposure on non-hepatic targets, such as significant changes found in their microbiomes. Thus, to validate the direct effects of NNS on liver toxicity, we investigated the direct exposure to NNS on liver cells to determine their impact on liver health and detoxification.

**Figure 1:**
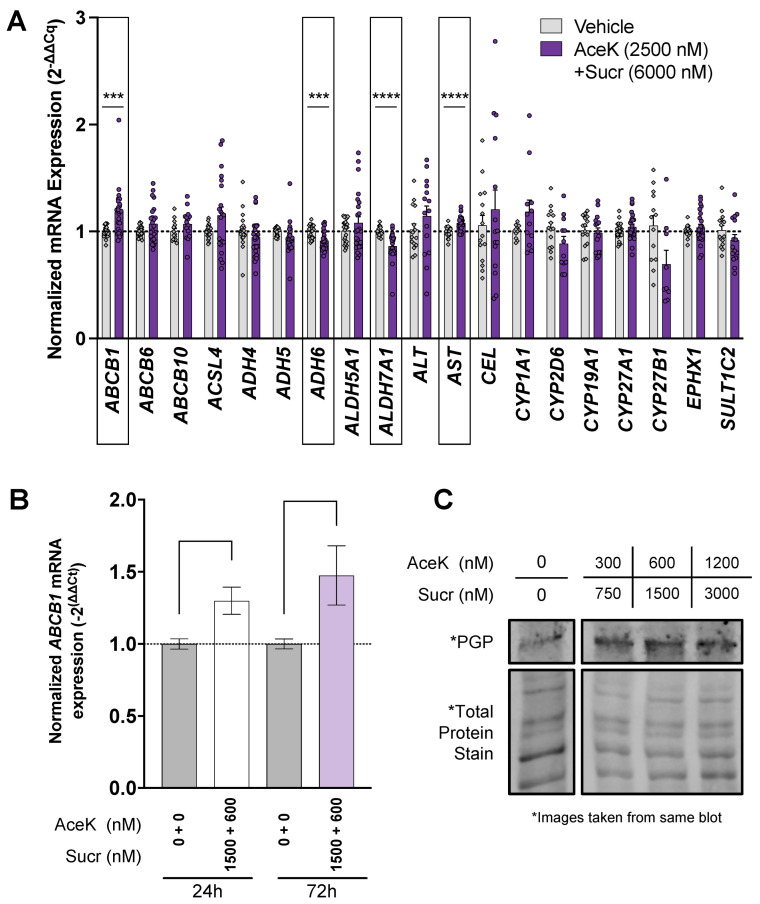
**Acesulfame potassium (AceK) and sucralose (Sucr) alter expression of liver enzymes and P-glycoprotein.** (**A**) HepG2 liver carcinoma cells were treated with a mixture of AceK and Sucr at a concentration equal to plasma concentrations after consumption of the acceptable daily intake (ADI) for each sweetener (AceK 2500 nM + Sucr 6000 nM). Expression of liver detoxification mRNA transcripts was measured by RT-qPCR and normalized against beta-actin (*ACTB*). Significance was assessed by multiple unpaired t-tests. (**B**) HepG2 were treated with AceK and Sucr at concentrations found in human plasma after consumption of an NNS-containing diet beverage (AceK 600 nM + Sucr 1500 nM) for either 24 h or 72 h. *ABCB1/PGP* expression was measured by RT-qPCR and normalized against beta-actin (*ACTB*). Significance was assessed by ordinary one-way ANOVA. (**C**) HepG2 were treated with a range of concentrations of combined AceK + Sucr for 72 h. PGP expression was measured by Western blot and normalized by Total Protein Stain (Invitrogen). * *p* < 0.05; *** *p* < 0.001; **** *p* < 0.0001.

HepG2 liver carcinoma cells were treated with a mixture of AceK and Sucr at a concentration equal to plasma concentrations following ingestion of the FDA-approved acceptable daily intake (ADI) for each sweetener (AceK 2500 nM + Sucr 6000 nM), which replicates the amount of NNS exposure in plasma to mice fed NNS daily in the previous study [6] (Figure 1A). Expression of liver health and detoxification gene mRNA transcripts was measured by RT-qPCR and normalized against beta-actin (*ACTB*). NNS treatment for 24 h significantly decreased the expression of alcohol dehydrogenase 6 (*ADH6*) and alcohol dehydrogenase 7A1 (*ALDH7A1*), while increasing the expression of aspartate aminotransferase (*AST*) and *ABCB1*, the gene encoding P-glycoprotein (PGP), henceforth labeled *ABCB1/PGP* (Figure 1A). Interestingly, in rat colon, increased *ABCB1/PGP* expression was also reported following the sucralose diet [16].

Thus, we further investigated the impact of NNS on *ABCB1/PGP* expression in human liver-origin cells. HepG2 cells were treated with a combination of AceK and Sucr at concentrations found in human tissues after consumption of one diet soda (Table 1). Cells were treated for 24 or 72 h, and *ABCB1/PGP* transcripts were measured by RT-qPCR. *ABCB1/PGP* mRNA levels were significantly increased following combined NNS exposure at both timepoints (Figure 1B). We next examined whether treating HepG2 with NNS would consequently alter PGP protein levels. Because PGP has a long half-life (~27 h) [19], we treated HepG2 with combined AceK and Sucr for 72 h and probed PGP levels by Western blot. NNS treatment led to increased PGP levels (Figure 1C), consistent with reports of increased PGP levels following treatment with substrate drugs and inhibitors [34].

### 3.2. Acesulfame Potassium and Sucralose Inhibit Efflux of PGP Substrates

**Figure 2:**
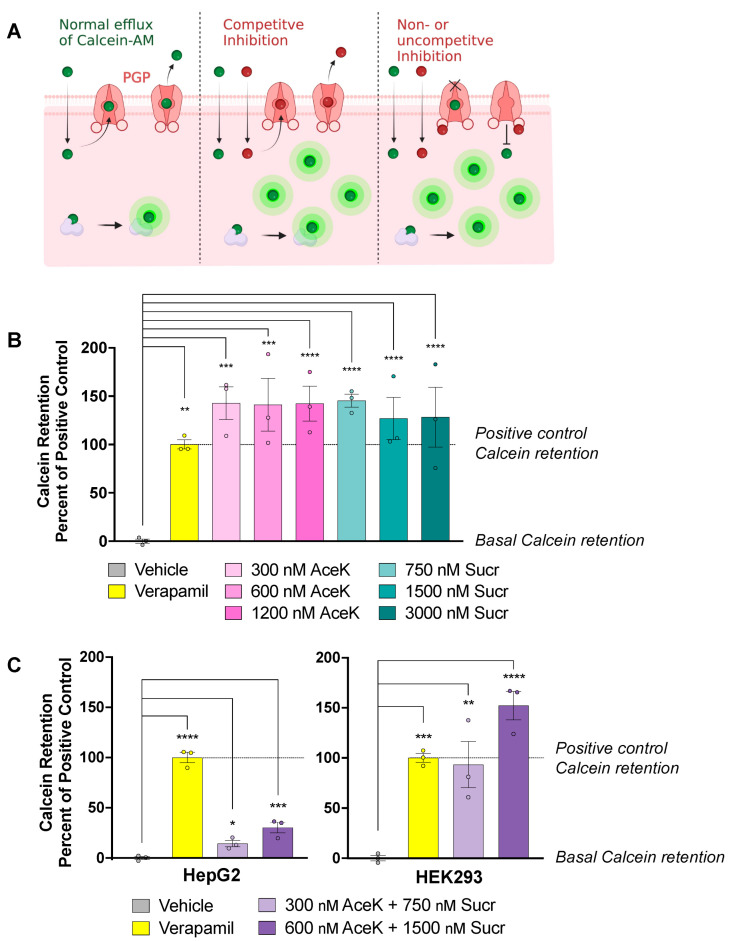
**Acesulfame potassium (AceK) and sucralose (Sucr) inhibit PGP substrate efflux**. (**A**) PGP inhibition can be measured in vitro through an assay of Calcein-AM retention. Calcein-AM is a pre-fluorescent compound and substrate of PGP. Under basal conditions, it is continually effluxed by PGP. In the presence of an inhibitor compound, Calcein-AM is trapped within the cell, where non-specific esterases convert it to its fluorescent metabolite Calcein. Created with BioRender (**B**) HepG2 were incubated with individual AceK or Sucr, 50 μM Verapamil, or vehicle control. PGP inhibition was measured as increased fluorescent signal from Calcein. Significance was assessed by ordinary one-way ANOVA. (**C**) HepG2 and HEK-293 were incubated with Calcein-AM, along with positive control 50 μM Verapamil or combined NNS. Significance was assessed by ordinary one-way ANOVA. * *p* < 0.05; ** *p* < 0.01; *** *p* < 0.001; **** *p* < 0.0001.

*ABCB1/PGP* expression is frequently altered in response to a change in function. Indeed, vinca alkaloid PGP substrates such as Vincristine have been shown to increase *ABCB1/PGP* expression in response to its competitive inhibition [35]. To understand whether NNS inhibit the efflux of PGP substrates, we performed fluorescence-based PGP inhibition assays (Figure 2A). Calcein-AM is a substrate for PGP which, under normal conditions, is continually effluxed out of cells by PGP in a cyclical fashion. When treated alongside a potent PGP inhibitor such as Verapamil, Calcein-AM is retained intracellularly due to competitive, non-competitive, or uncompetitive PGP inhibition. Cytosolic esterases within the cell then convert trapped Calcein-AM into its fluorescent metabolite Calcein, which cannot be transported by PGP. Thus, PGP inhibition is represented by an increased fluorescent signal.

As our primary focus is understanding the effects of NNS consumption on liver detoxification, hepatic cells were selected for our initial efflux inhibition studies. HepG2 cells were treated with a range of concentrations of individual AceK and Sucr, and Calcein retention was measured by the resulting fluorescent signal intensity. Verapamil, a calcium channel blocker and potent competitive inhibitor of PGP, was used as a positive control for PGP inhibition [36,37]. AceK and Sucr treatment led to significant increases in Calcein retention, thus indicating inhibition of Calcein-AM efflux (Figure 2B). Inhibition of Calcein-AM efflux was observed at AceK and Sucr concentrations as low as 300 and 750 nM, respectively. These are well within concentrations expected in human plasma after drinking one NNS-sweetened beverage (Table 1).

While HepG2 are a useful model for human liver cells, they express abundant levels of other related ABC transporters such as MRP2. Both Calcein-AM and Calcein are substrates for MRP2 [38,39,40], thus creating a doubt as to which transporters Calcein is expelled by in these cells and, thus, which transporter is inhibited by NNS. On the other hand, HEK-293 cells generated from a human embryonic kidney express high endogenous levels of PGP while exhibiting negligible levels of MRP2 [41]. Therefore, in these cells, Calcein efflux can be attributed to PGP activity. We treated both HepG2 and HEK-293 cells with a combination of AceK and Sucr and measured the fluorescent signal from Calcein retention (Figure 2C). Interestingly, Calcein retention was more pronounced in HEK-293 cells than in HepG2, suggesting specificity for NNS inhibition to PGP.

### 3.3. Sucr and AceK Stimulate PGP Efflux Activity

**Figure 3:**
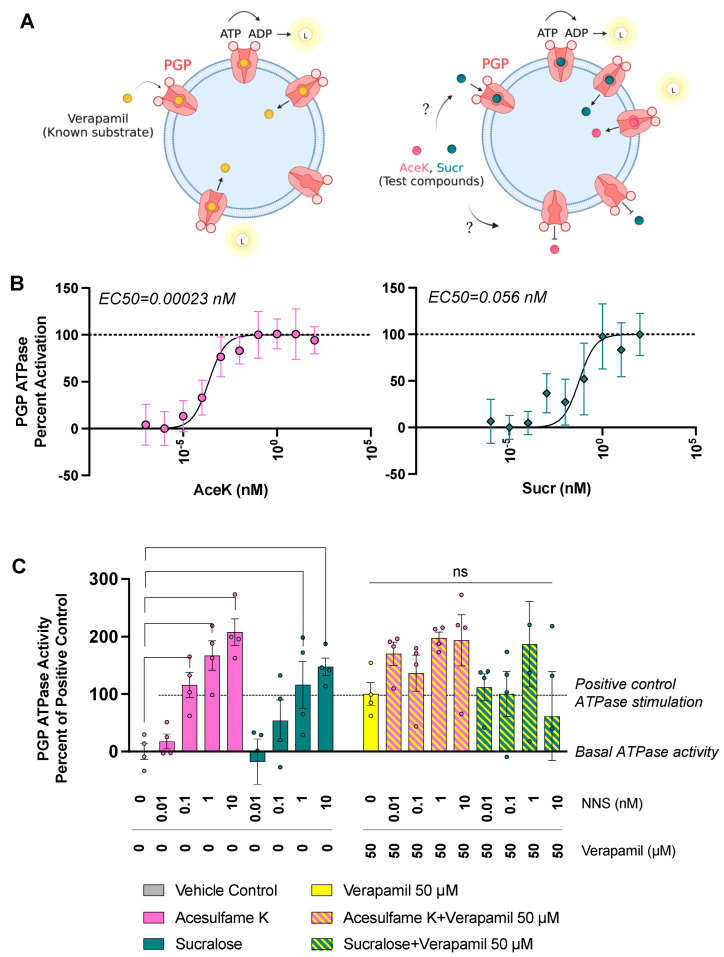
**AceK and Sucr stimulate PGP ATPase activity. (A) ATP hydrolysis is required for the conformational change that accomplishes substrate efflux.** PGP ATPase activity can be assessed in a cell-free assay. Vesicles containing human PGP are treated with compounds known to stimulate ATP hydrolysis and test compounds. Created with BioRender (**B**) PGP membranes were treated with increasing concentrations of AceK or Sucr. Ec50 values were determined by non-linear fitting of [Agonist] vs. response (95% CI: AceK, 6.074 × 10^−5^ to 0.001089 nM; Sucr, 004922 to 0.4787 nM). (**C**) AceK and Sucr were incubated with PGP membranes individually and in the presence of 50 μM Verapamil. Verapamil, a known substrate of PGP, leads to strong stimulation of ATPase activity alone. AceK and Sucr individually stimulate PGP ATPase activity in a dose-dependent manner. Co-incubation of AceK or Sucr with Verapamil does not significantly increase or decrease ATPase stimulation relative to Verapamil alone. Significance was assessed by ordinary one-way ANOVA.

Many PGP inhibitors, including Verapamil, are also high-affinity substrates for PGP [24]. Thus, they prevent the efflux of other substrates, such as Calcein-AM, by binding PGP with higher affinities. The transport of these high-affinity competitive inhibitors requires ATP consumption, which catalyzes the conformational shift associated with the substrate efflux [42] (Figure 3A). PGP in the substrate-bound conformation is inward-facing, while the outward-facing conformation allows substrate release. Therefore, measuring ATP consumption by PGP is a common method for determining the binding kinetics of putative substrates and competitive inhibitors [43,44]. Interestingly, organochlorine compounds, the family that includes Sucr, are often PGP substrates [10]. AceK, however, as a highly water-soluble potassium salt, does not fit the typical profile for PGP substrates.

To verify if Sucr and AceK are substrates of PGP and act as competitive inhibitors, we measured PGP ATPase activity. Measurement of ATP consumption on artificial lipid vesicles exclusively containing recombinant human PGP provides a minimal system to assess PGP activity. Individual AceK and Sucr at a range of concentrations were incubated with PGP membranes. ADP produced by the hydrolysis of ATP by the ATPase domains of PGP was measured by ADP-Glo Max assay (Promega, Madison, WI, USA), which utilizes a luciferase/luciferin reaction to produce an ADP-dependent luminescent signal (Figure 3A). Both AceK and Sucr increased ATPase activity in a dose-dependent manner, with EC50 values for AceK and Sucr being 0.00023 nM and 0.056 nM, respectively (Figure 3B). Thus, AceK and Sucr are effluxed by PGP and are substrates of this transporter. We therefore demonstrated that AceK and Sucr are transported by PGP at concentrations within expected plasma levels after the ingestion of one NNS-sweetened beverage (Table 1), suggesting that they are competitive inhibitors of PGP.

We next confirmed that the inhibitory effect of Sucr and AceK on PGP was through competitive and not allosteric inhibition. If Sucr and AceK were also acting as allosteric inhibitors, their co-incubation with a competitive inhibitor such as Verapamil would lead to decreased PGP activity. Thus, AceK and Sucr were incubated with PGP membranes supplemented or not with 50 μM Verapamil. As expected, Verapamil alone significantly stimulates ATPase activity (Figure 3C). As previously observed, individual AceK and Sucr treatments stimulated PGP ATPase activity, confirming NNS transport in a dose-dependent manner. However, co-incubation of Verapamil with AceK or Sucr did not lead to significant changes in PGP activity relative to Verapamil alone, demonstrating that they do not inhibit PGP allosterically in the presence of potent substrates.

### 3.4. AceK and Sucr Show Unique PGP Binding Patterns In Silico

**Figure 4:**
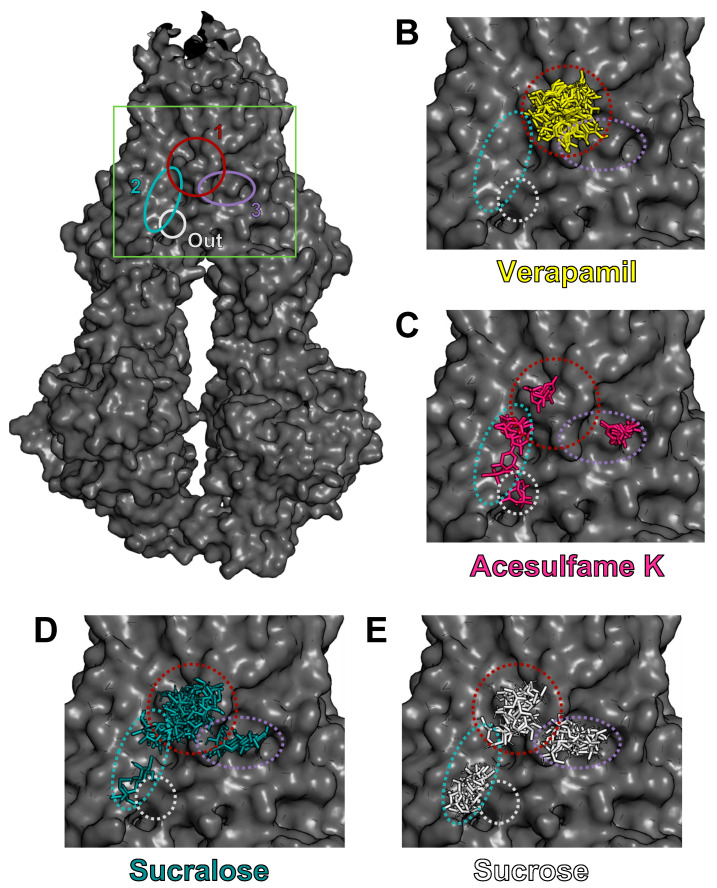
**Molecular docking experiments show AceK and Sucr in PGP binding pockets.** Docking experiments were performed with AutoDock Vina and figures were prepared with PyMOL. Structure for full-length PGP retrieved from RCSB Protein Data Bank (PDB: 7A65). 3D structures for Verapamil, AceK, and Sucr retrieved from ZINC. (**A**) Structure of human PGP from cryo-EM (PDB: 7A65) showing the location of main binding pockets in transmembrane region. (**B**–**E**) Insert of transmembrane region showing PGP with Verapamil (**B**), AceK (**C**), Sucr (**D**), or Sucrose (**E**) docked in binding pockets. Binding pockets are shown as: Pocket 1:burgundy; Pocket 2:indigo; Pocket 3:teal; External binding site:gray.

**Figure 5:**
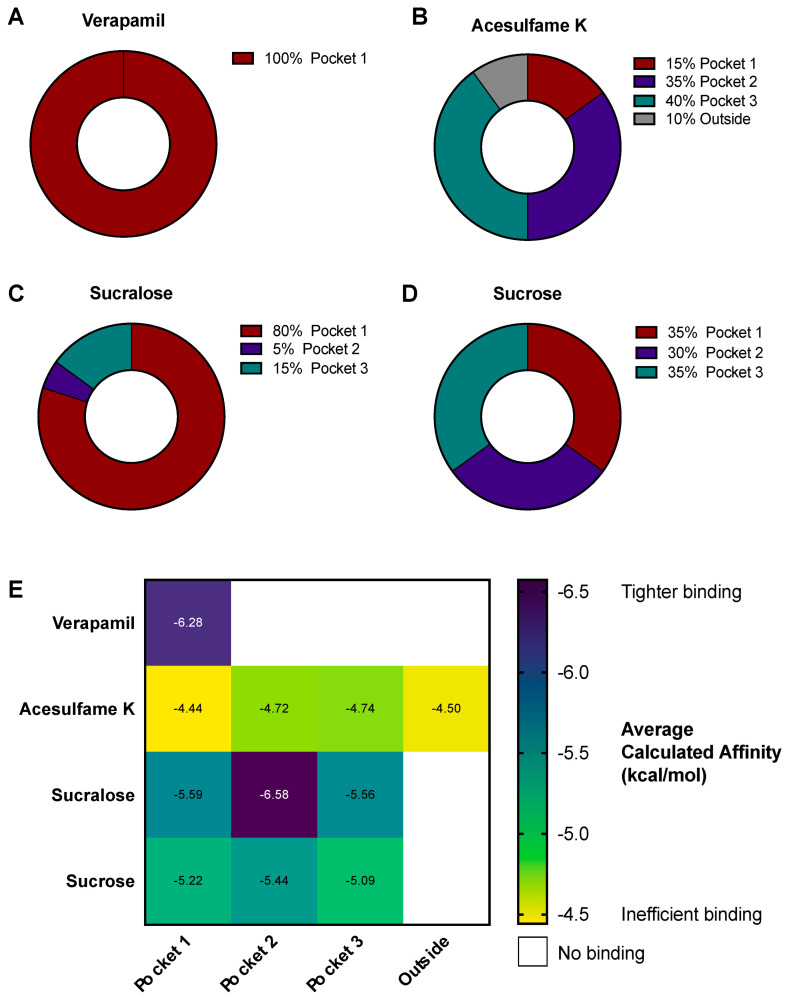
**Binding pocket occupancy for compounds docked on PGP.** All docking poses for each compound were visualized on PyMOL and inventoried according to their position in PGP transmembrane binding pockets. (**A**–**D**) Frequency of poses in each binding pocket for each docked compound. (**E**) Average calculated binding affinity for each compound and binding pocket combination. Tighter binding indicated by indigo and weaker, inefficient binding indicated in yellow. Compound/binding pocket combinations that were not found among top 20 docking poses are shown in white.

To further explore the interactions between NNS and PGP and elucidate their inhibitory mechanism, we performed virtual docking experiments of AceK and Sucr in the PGP transmembrane domain, which includes multiple cavities, i.e., pockets, in which substrates bind before efflux (Figure 4A, green box). Previously acquired crystal structures of PGP in complex with inhibitors, such as Vincristine, demonstrated that competitive inhibitors mainly bind in a flexible, hydrophobic pocket within the transmembrane domain [24,45] (Figure 4A, burgundy “pocket 1”). Docking of Verapamil was also performed as a positive control for high-affinity binding, and sucrose (table sugar) was also used in docking experiments as a compound structurally similar to sucralose. Indeed, sucrose lacks three chloride substitutions and is ideal for assessing the specificity of sucralose binding to PGP. As expected, Verapamil docked solely in the high-affinity binding pocket (Figure 4B and Figure 5A). AceK showed a lower preference for binding within the high-affinity pocket and docked in multiple locations within the transmembrane domain (Figure 4C and Figure 5B). In contrast, Sucr docking positions were mostly found within the same binding pocket as Verapamil (Figure 4D and Figure 5C). Furthermore, despite the high structural overlap, Sucrose showed no preference for PGP pockets (Figure 4E and Figure 5D), suggesting Sucr had a preference for the high-affinity binding pocket of PGP due to its chloride groups. A full report of docking positions, along with calculated metrics, including binding affinities and root mean square deviation (RMSD), can be found in Table S2. As expected, Verapamil showed the highest calculated binding affinity of all tested compounds, below −6.5 kcal/mol (Figure 5E). Sucr generally showed higher average calculated binding affinities for each pocket than sucrose or AceK (Figure 5E). Calculated binding affinities for AceK were relatively low across all regions in which it docked on PGP, which included two poses outside the transmembrane channel (Figure 5E).

**Figure 6:**
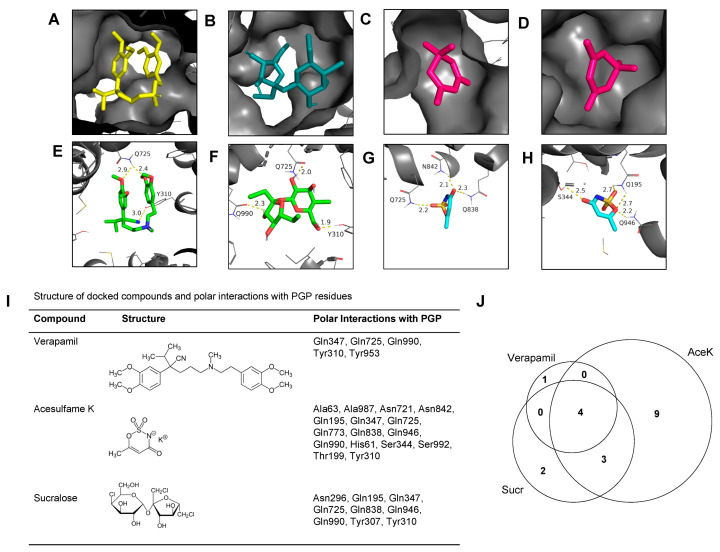
**AceK and Sucr make similar amino acid contacts in binding pockets as known PGP substrates.** (**A**–**D**) Select docking poses showing cartoons of Verapamil (**A**), Sucr (**B**), and AceK (**C**,**D**) nested in PGP binding pockets on PyMOL. PGP structure 7A65 retrieved from RCSD Protein Data Bank. Docked compounds retrieved from ZINC database. (**E**–**H**) Polar contacts for select poses with labeled PGP amino acid residues for Verapamil (**E**), Sucr (**F**), and AceK (**G**,**H**). Dashed yellow lines indicate polar bonds and bond lengths are given in Angstroms. (**I**) Structures for each docked compound are provided along with PGP amino acids residues found in polar contacts for each. In agreement with the literature on PGP substrates, residues contacted by all compounds in common include Gln347, Gln725, Gln990, and Tyr310. (**J**) Venn diagram illustrating overlaps between PGP residue polar contacts for each docked compound. Verapamil shares all but one polar contact with both AceK and Sucr. Sucr shares most polar contacts with Verapamil and AceK, with only two unique amino acid contacts. AceK has the most divergent binding profile with both the greatest total polar contacts and the most unique polar contacts.

Polar contacts for all docking poses were also inventoried for Verapamil, Sucr, and AceK (Figure 6). In agreement with the literature on PGP substrates [30,31,32], residues contacted by all compounds in common include Gln347, Gln725, Gln990, and Tyr310 (Figure 6I). The distribution of polar contacts in the PGP binding pockets between each compound is further illustrated in Venn diagram format (Figure 6J) and showed that in contrast to AceK, Verapamil and Sucr share most amino acid contacts with PGP.

## 4. Discussion

Initially characterized in 1976 [46], P-glycoprotein (PGP) is an essential mediator of drug metabolism and poses a challenge in treating cancers by causing multidrug resistance in tumors, which gives PGP its alternative name Multidrug Resistance Protein 1 (MDR1). Furthermore, PGP has many endogenous substrates and participates in the distribution of steroid hormones, biliary pigments, cytokines, and short-chain lipids [47,48,49,50]. Due to the substrate overlap with other members of the ATP-binding cassette transporter superfamily, deletion of PGP is non-lethal in animal models [51,52]. However, the absence of PGP, or mutations in *ABCB1*, the gene encoding PGP, can dramatically alter the pharmacokinetics of PGP substrates [53]. For example, because PGP is expressed at the maternal–fetal interface, mutations on the gene encoding PGP can lead to lower placental PGP expression and increased fetal exposure to drugs or environmental pollutants during pregnancy [54,55,56,57,58,59]. Similar effects can also be observed when PGP is pharmacologically inhibited. For instance, a higher incidence of birth defects was reported among infants born to mothers who took PGP substrate or inhibitor drugs during pregnancy, presumably resulting from higher fetal exposure to teratogens [60]. Additionally, altered PGP expression or PGP inhibition contributes to altered pharmacokinetics and drug–drug interactions [61]. In an era where many common prescription drugs are PGP substrates, including antidepressants (amitriptyline, citalopram, sertraline), antibiotics (levofloxacin, erythromycin), antivirals (saquinavir, ritonavir), and more [17,60,62], it is essential to know which compounds inhibit or stimulate PGP to prevent toxicity and improve treatment efficacy. Particularly, dietary compounds, such as non-nutritive sweeteners (NNS), have been suggested to be PGP substrates [10,35].

Commonly consumed in both Western and Asian countries, NNS are increasingly present in food, beverages, and other products that are marketed as “diet” or “zero” options, as well as many with no such indications [4,63,64]. By increasing palatability at a low cost and without raising overall calories, NNS are highly attractive to manufacturers. However, consumers are not able to reliably identify products that contain NNS and may be ingesting them regularly without knowing it [65]. It is estimated that over 40% of the adult population in the United States consumes NNS [3]. Additionally, prescription drug use in the United States continues to rise, and many of these medications are PGP substrates. Prescription drug use includes about 13% of adults in the United States taking antidepressants, over 80% of patients in the United Kingdom or 40% of patients in the United States with type 2 diabetes mellitus prescribed metformin, and up to 80% of people in the United States taking an antibiotic prescription within the course of a year [66,67,68,69]. Therefore, the potential of drug–NNS interactions is intriguing.

Amongst the NNS that are approved for consumption by the Food and Drug Administration (FDA) and the Joint FAO/WHO Expert Committee on Food Additives (JECFA), sucralose (Sucr) and acesulfame potassium (AceK) are now used more frequently due to a decline in popularity for earlier sweeteners such as saccharin and aspartame. Along with their predecessors, these two NNS have also come under scrutiny for their potential health risks. A large population-based study recently linked high rates of AceK consumption with increased risks of breast and metabolic-related cancers [70]. Previously, sucralose has been linked to both dysregulation of the microbiome and increased PGP levels in the large intestine of rats [16]. Further study into the impact of NNS on PGP was necessary to elucidate the mechanisms for this interaction. Importantly, for the first time, we investigated the effects of combined AceK and Sucr on PGP, which more closely replicates real-world exposures from food and beverage products that frequently contain combined sweeteners (Gatorade G2, Powerade Zero, Diet Mountain Dew). Furthermore, our experiments modeled the exposure to AceK and Sucr in simplified systems, showing a direct link between NNS and PGP dysregulation. Our results also showed that longer NNS exposure times (~24–72 h) increased *ABCB1*/PGP expression, while acute exposure (~30 min) caused competitive inhibition of PGP efflux function. This shows that NNS consumption may have unexpected consequences even in short-term situations such as drinking an occasional diet soda.

A recent study from our lab also suggested the potential inhibition of PGP by NNS [6]. Mouse pups from mothers fed NNS during pregnancy and lactation showed decreased plasma levels of endogenous ATP-binding cassette transporter substrates, including PGP substrates bilirubin and biliverdin [6]. This suggested a failure of hepatic PGP to regulate proper dispositions of unconjugated bile pigments. We posit that the NNS exposure from the mothers’ diets inhibited natural PGP efflux, leading to the accumulation of PGP substrate compounds and liver toxicity, evidenced by metabolomics data and gross whitening of the pups’ livers. Due to the design of this previous study, however, we were not able to make this claim conclusively. The NNS exposure in the study in mice was not explicitly targeted to the liver, and indeed, significant dysregulation of pups’ microbiome was also revealed, preventing us from concluding that NNS actions on hepatic PGP alone led to the liver dysfunction. Additionally, those experiments only used mixed AceK and Sucr, preventing the determination of the role of individual NNS in PGP deregulation. Finally, NNS exposure in mice began at conception and continued through lactation, a period totaling roughly 40 days. As such, the effects of such long-term exposure on hepatic PGP may have extended beyond acute functional inhibition. This initial evidence that NNS might not only bind to PGP but also impact its activity was validated in the current study.

Here, we opted for a more targeted approach to directly assess the impact of NNS on PGP in liver cells and demonstrated that concentrations of AceK and Sucr found in human plasma after drinking one NNS-sweetened beverage competitively inhibit PGP function. These effects would be present both when consumed individually (e.g., Splenda sweetener packets—Sucr only, Diet Coke—AceK only) or combined (e.g., Gatorade G2, Powerade Zero, Diet Mountain Dew). Despite a lack of evidence for the safety of combinations of food additives, NNS are frequently combined in food and beverage products because of their complementary effects on taste. A deeper exploration of NNS combinations is therefore warranted and is especially relevant in the detoxification pathways, which utilize transporters with wide substrate recognition.

Prior to this study, Sucr had already been suggested to be a substrate of PGP due to its structure as an organochlorine molecule, many of which are PGP substrates [10]. Our study confirmed Sucr as a substrate through functional assays and in silico docking experiments and emphasizes that Sucr enters the transmembrane channel of PGP and binds within efflux-stimulating substrate pockets. Sucr docks mainly in the same high-affinity, high-turnover pocket as Verapamil, a known potent PGP substrate. AceK, a structurally dissimilar NNS not previously implicated as a PGP substrate, shows similar impacts on PGP function as Sucr experimentally. AceK effectively inhibits Calcein-AM efflux and stimulates PGP ATPase function at concentrations equal to or lower than Sucr, respectively. In docking experiments, AceK shows more promiscuous interactions within the transmembrane region while still being capable of making polar contacts with key amino acid residues that trigger efflux. The binding of Sucr and AceK into PGP suggests that they might compete with PGP substrates and act as inhibitors at low picomolar concentrations.

Based on our cellular efflux assay, we imagine that when AceK and Sucr are consumed along with PGP substrate medications, they may be preferentially effluxed by PGP, leading to the retention of other PGP substrates, altered drug distribution, and increased cellular toxicity. As novel PGP inhibitors, it is crucial to determine the extent of the NNS–drug interactions to understand how they may impact pharmacological therapeutic interventions throughout the body. This is not only critical in the liver, but in all tissues expressing PGP. The activity of PGP at blood–tissue barriers has been studied extensively, particularly at the intestine, liver, kidneys, and blood–brain barrier. Unaccounted functional inhibition of PGP at any of these sites could lead to higher rates of drug exposure than intended and compromise therapeutic efficacy [71,72,73]. Adverse effects of such drug-drug and drug–food interactions have been documented for decades, including prior to the identification of PGP as the transporter responsible for this phenomenon [74,75]. For example, increased plasma levels of orally administered digoxin or fexofenadine were observed after co-administration of Verapamil, a calcium channel blocker used as an anti-hypertensive and now widely known as a PGP competitive inhibitor [36,74,75]. Many PGP-mediated food–drug interactions are known, including the disruption of PGP substrate drug uptake by components of grapefruit juice and soybean products [71,72,73,76]. Although knowledge of such interactions is available to prescribing physicians, a recent study reported that patient counseling may be inadequate [77]. Risks of interactions with foods and herbal supplements may be greater as they are freely available without prescriptions. Herbal remedies are more likely to be considered “safe” by consumers, despite potentially having multiple biologically active compounds [78]. To our knowledge, our studies are the first to show that the NNS AceK and Sucr may belong among this group of foods that interact with PGP substrate drugs. Further study is necessary to uncover the extent of the potential of AceK and Sucr to interfere with the expected absorption and distribution of PGP substrate drugs. This will be of particular interest to populations that may have a higher risk of exposure to both NNS and PGP substrate drugs. In Western countries, women and people with type 2 diabetes are known to consume NNS at higher rates [3]. Among people taking PGP substrate drugs, women are more likely to take certain antidepressants [66] or metformin for reproductive conditions [79].

While our experiments present initial evidence of AceK and Sucr deregulation of PGP, some limitations prevent drawing conclusions about clinical risks at this time. Our in vitro experiments on *ABCB1* and PGP expression utilized HepG2, an established cell line of human liver cancer origin. While HepG2 cells endogenously express functional PGP, primary human hepatocytes or more advanced model systems such as organoids would provide important physiological evidence of NNS deregulating PGP expression in healthy liver tissue. Furthermore, our experiments did not test the effects of NNS on the efflux of other PGP substrate drugs. Thus, future experiments should assess the impact of NNS on the transport of PGP substrate drugs in vitro and in vivo, ideally in multiple tissue types where PGP plays a critical role in drug distribution (e.g., intestine, blood–brain barrier, placenta) [17]. This will improve our understanding of how dietary NNS exposure could interfere with therapeutic interventions. Future studies should address whether the dietary consumption of AceK and Sucr pose challenges in complex health conditions where PGP activity is relevant such as advanced liver diseases or certain cancers. Furthermore, future work should investigate the impacts of NNS on PGP-mediated drug transport during pregnancy, where PGP plays a critical role in protecting the developing fetus from toxic exposures [60,80,81].

## 5. Conclusions

Using a combination of human hepatic- and kidney-origin cells, cell-free biochemical assays, and molecular docking, we have demonstrated that sucralose and acesulfame potassium act as competitive inhibitors of P-glycoprotein at concentrations as low as levels found in human plasma after drinking one non-nutritive sweetener (NNS)-sweetened beverage. Thus, previous findings from NNS-fed mice and our present results support a role for NNS in impairing proper PGP function and cellular detoxification. Due to the crucial role of PGP in various tissues, including drug absorption, xenobiotic defense and clearance, and distribution of endogenous substrates such as steroid hormones and cytokines, it is imperative to fully characterize the consequences of sucralose and acesulfame potassium exposures among target populations. Future work must elucidate the impact of these popular NNS on the absorption and clearance of common PGP substrate drugs in relevant tissue types, including the intestine, blood–brain barrier, and placenta. Clinical studies will reveal whether NNS consumption poses additional risks to patients prescribed PGP substrate drugs. By enhancing our fundamental understanding of the biological effects of NNS, we hope to improve the guidelines for food consumption during pharmacologic interventions and prevent harmful behaviors in patients.

## Figures and Tables

**Table 1:** **Acesulfame potassium (AceK) and sucralose (Sucr) concentrations revealed in tissues following dietary exposure.** AceK and Sucr are absorbed into the bloodstream following dietary exposure and circulate intact as they bathe tissues in the body [1]. References giving concentrations for each sweetener in different tissues were selected and are shown here with their highest recorded concentrations.

Acesulfame Potassium	Max Concentration ng/mL (nM)
Human plasma [22]	1500 (7500)
Human breastmilk [21]	1000 (5000)
Mouse plasma [6]	250 (1250)
**Sucralose**	**Max Concentration ng/mL (nM)**
Human plasma [20]	300 (750)
Human breastmilk [21]	1200 (3000)
Mouse plasma [6]	80 (200)

## Data Availability

All data are available in the main text or the Supplementary Materials.

## Supplementary Materials

The following supporting information can be downloaded at: https://www.mdpi.com/article/10.3390/nu15051118/s1, Figure S1: Combined AceK + Sucr treatment increases PGP exposure in HepG2; Figure S2: Calcein retention measured at different timepoints for varying Calcein-AM concentrations; Figure S3: Sensitivity of human cell lines to Calcein-AM efflux inhibition; Figure S4: PGP ATPase stimulation by Verapamil; Table S1: Gene primers used in HepG2; Table S2: Log files from Autodock VINA (ADV) molecular docking experiments and manually assigned pocket designation.
