# Supplementary material for: Non-Nutritive Sweeteners Acesulfame Potassium and Sucralose Are Competitive Inhibitors of the Human P-glycoprotein/Multidrug Resistance Protein 1 (PGP/MDR1)
Source: Nutrients. 2023 Feb 23;15(5):1118. doi: 10.3390/nu15051118 (PMC10005754; doi:10.3390/nu15051118)
Supplement: Supplementary file 1 [file nutrients-15-01118-s001.zip › nutrients-2223254-supplementary.pdf]

Supplementary Material

A Full blot – anti-PGP, no annotation

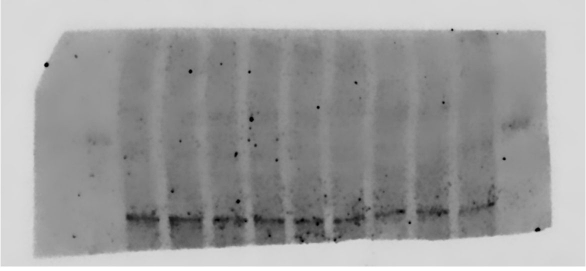

B Full blot – anti-PGP, with annotation

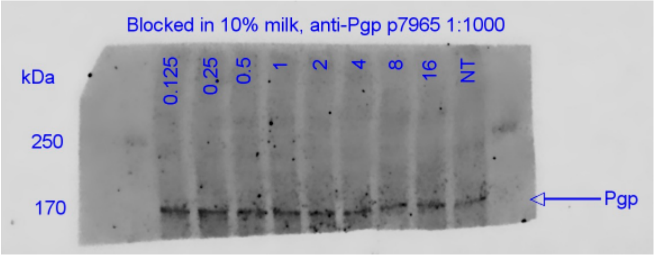

C Full blot – Total Protein Stain, no annotation

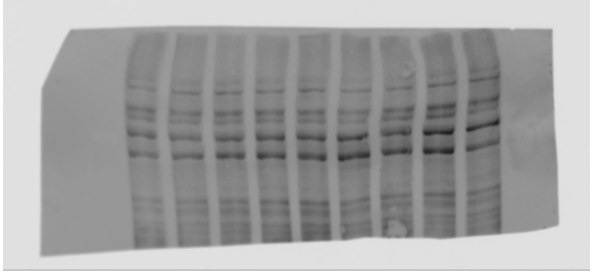

D Full blot – Total Protein Stain, with annotation

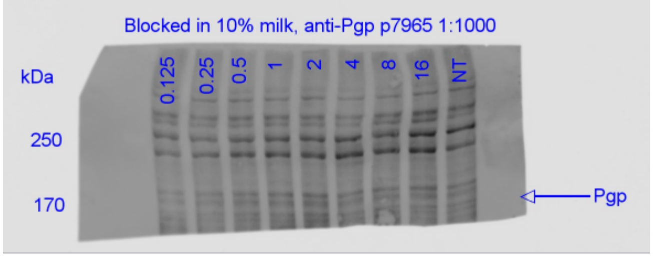

E Conversions for AceK and Sucr concentrations

NNS x ADI	AceK µM	+	Sucr µM
0.125	300		750
0.25	600		1500
0.5	1200		3000
1	2400		6000
2	4800		12000
4	9600		24000
8	19200		48000
16	38400		96000

Figure S1. Combined AceK + Sucr treatment increases PGP exposure in HepG2. (**A-D**). Full western blot images for blot seen in Figure 1. (**A)** and (**B)** show full blot with anti-PGP antibody p7965 from Sigma Aldrich. Annotations show protein size to the left and lanes are labeled with concentration of NNS treatment for HepG2. (**C)** and (**D)** show Total Protein Stain (Invitrogen) for full blot. (**E**) Conversion table shows concentrations of AceK and Sucr. Left column gives combined AceK + Sucr concentration as multiples of the Acceptable Daily Intake (xADI) for each sweetener (e.g., 1xADI equals 1xADI for AceK + 1xADI for Sucr. Columns to the right show equivalent concentrations given in µM concentrations, which are consistent with values given through the manuscript.

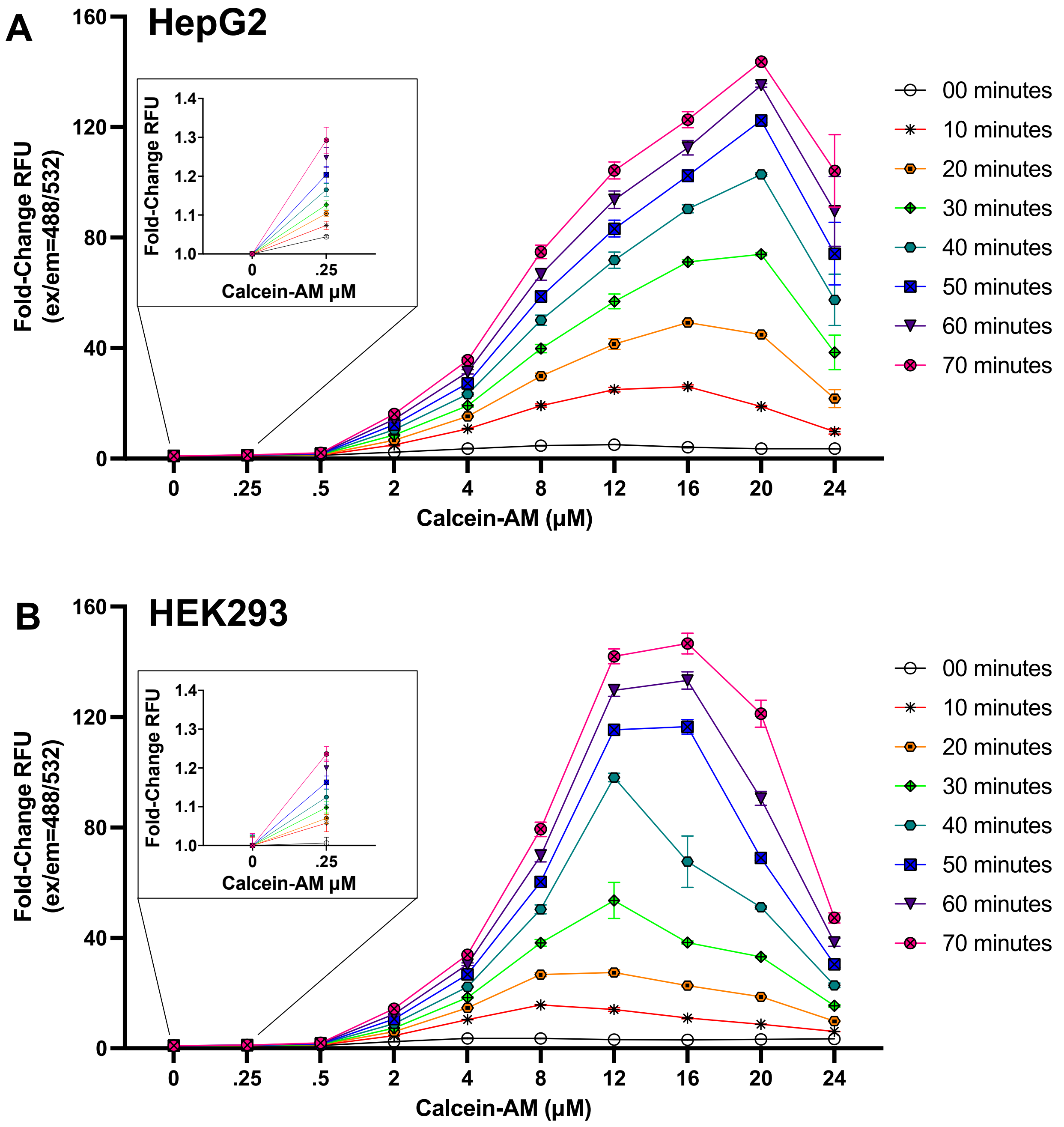

Figure S2. Calcein retention measured at different timepoints for varying Calcein-AM concentrations. (**A/B**) HepG2 (**A**) or HEK293 (**B**) were plated in clear 96-well plates and grown to 80-90% confluence. Cells were treated with varying concentrations of Calcein-AM and relative fluorescence was recorded every 10 minutes. Based on these results, conditions of 0.25 μM Calcein-AM treatment and 30-minute incubation were selected for Calcein retention experiments conducted without commercial kit.

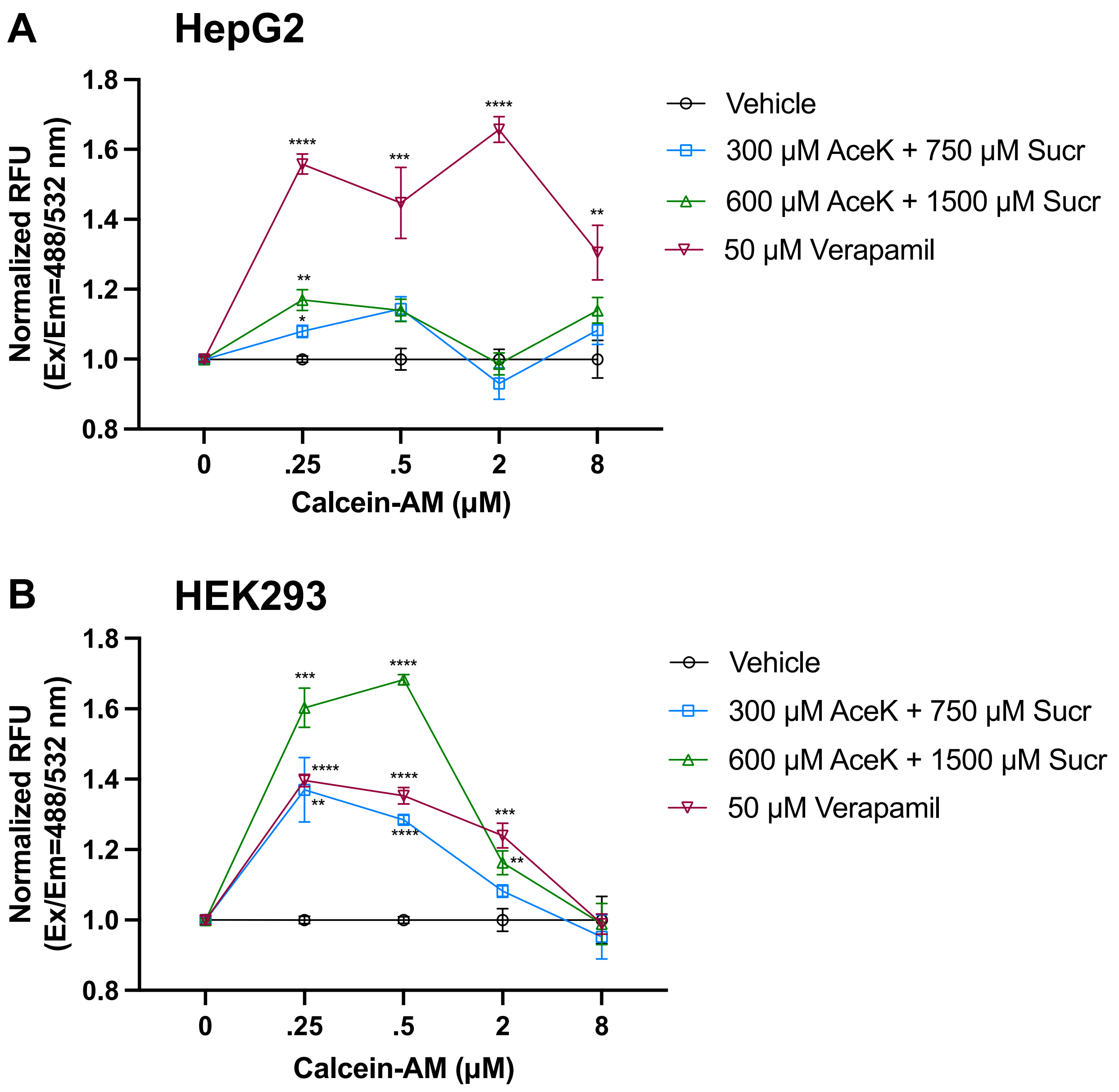

Figure S3. Sensitivity of human cell lines to Calcein-AM efflux inhibition. (**A**/**B**) HepG2 (**A**) or HEK293 (**B**) were plated in clear 96-well plates and grown to 80-90% confluence. Cells were treated with a combination of AceK + Sucr, Verapamil, or vehicle control, and then incubated with varying concentrations of Calcein-AM. Calcein-AM efflux inhibition was measured as fluorescence intensity of the fluorescent metabolite Calcein. Significance was measured as increased fluorescent signal based on treatment (NNS, Verapamil, vehicle) for each concentration of Calcein-AM.

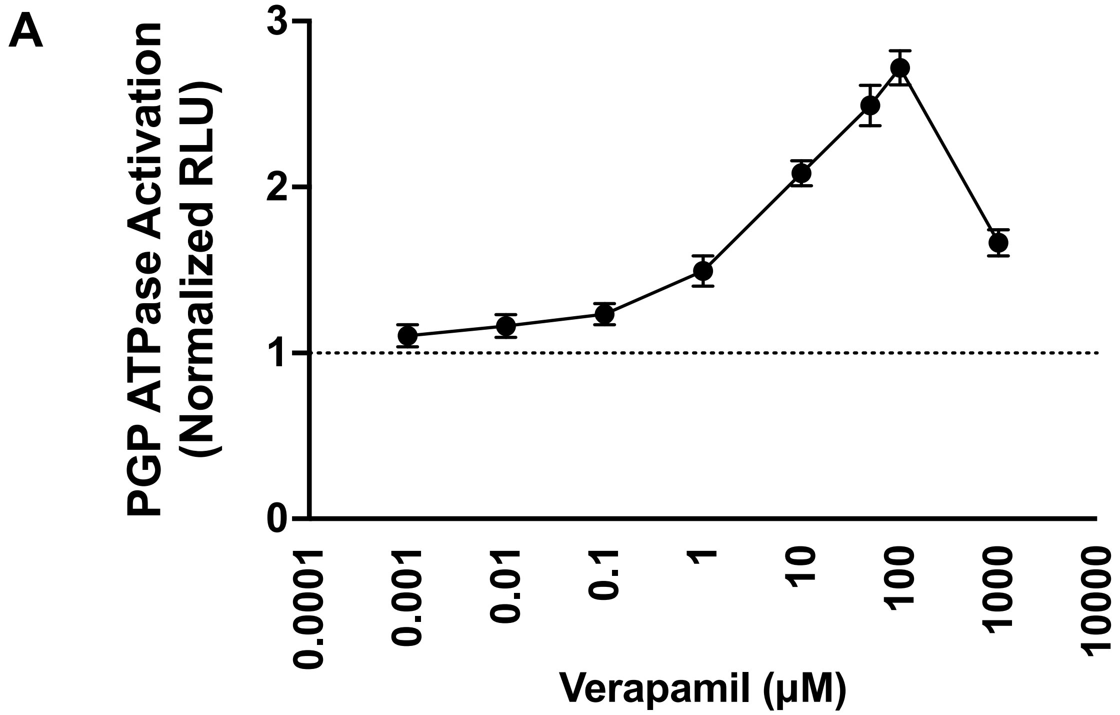

Figure S4. PGP ATPase stimulation by Verapamil. (**A**) PGP ATPase activity was monitored using the commercially available ADP-Glo Max Assay (Promega). Prior to treatment with AceK or Sucr test compounds, the assay system was validated using a range of concentrations of Verapamil, a known PGP substrate and competitive inhibitor. Peak ATPase activation at 50 and 100 µM Verapamil were in agreement with the literature [37].

Table S1. Gene primers used in HepG2.

Gene Name	Author	Size (bp)	Forward Seq 5' - 3'	Reverse Seq 5' - 3'	F Primer Tm	R Primer Tm
ADH4	Guo 2011	134	AGTTCGCATTCAGATCATT-GCT	CTGGCCCAATACTTTCCACAA	55	55.4
ADH5	Guo 2011	102	TGCCAGAAGATAAGAG-TCACTCA	GCTGGTTCCCATGTAATGCAA A	55.3	56.2
ADH6	Guo 2011	83	TGGTGCTGCAATCAATACTGC	CAACAGACAAGCCGACTCCT	56.1	57.2
ALDH5A1	Guo 2011	111	AGTCATCACCCCGTGGAATTT	GAGAAGGGCGTGTCTTCGG	56.6	58.1
ALDH7A1	Guo 2011	204	GATGATTGGAGGAC-CTATCTTGC	ACAGCCACAC-TAATGAGGGAA	55.1	56.1
CEL	Guo 2011	126	GTCACCTTCAACTACCGTGTC	GGCCGCGATATTCCTCTTCAC	55.3	58.1
CYP19A1	Lee 2007	94	AAGACGCAGGAT-TTCCACAGA	TCTTGTCAGGTCACCAC-GTTTC	56.5	57.2
CYP1A1	Lee 2007	84	GCTGCAACGGGTGGAATT	CAGGCATGCTTCATGGTTAGC	56.4	56.7
CYP27A1	Guo 2011	158	CAGCACGACCTGACCTATGG	TGGTCCAGTCGAG-TCATAAAGT	57.6	55.5
CYP27B1	Guo 2011	138	ACCAGATGTTTGCATTT-GCTCA	CAGGCAACTCTTCCCGGAAC	55.6	58.4
CYP39A1	Lee 2007	83	GGACCCATTACCCAAACAGA GTT	TTTGTTTATATTCAATTCGG-CATTG	57.1	51
ACSL4	Guo 2011	122	ACTGGCCGACCTAAGGGAG	GCCAAAGGCAAGTAGCCAAT A	58.9	55.7
EPHX1	Lee 2007	76	GGAGGCCTGGAAAGGAAGTT	TGATGGTGCCTGTT-GTCCAGTA	57.2	58.4
SULT1C2	Guo 2011	227	TCCTTTATGTAGCTCGAAATG CC	TGGGTCCCTCTTTATGTCCTC	55	55.9
ABCB1	Lee 2007	68	GTCCCAGGAGCCCATCCT	CCCGGCTGTTGTCTCCATA	59.4	56.9
ABCB6	Lee 2007	81	TTCAGAAGGGCCG-TATTGAGTT	TGAAAGACACGTCCTG-CAGAGT	56.3	58.4
ABCB10	Lee 2007	75	CCCCAAGGGTTCAACACTGT	AATCGCAATCCGCTGTTTCT	57.6	55.2
ALP(ALPL)	Olivier-Van Stich-elen Lab	159	AACATCAGGGACATTGAC-GTG	GTATCTCGGTTTGAA-GCTCTTCC	55.3	55.4
ALT (GPT)	Olivier-Van Stich-elen Lab	213	CCAGGGTGTGAAGAA-GCCTTT	CTGTAGGCCCCCAGACTGT	57.6	59
AST (GOT1)	Olivier-Van Stich-elen Lab	90	ATTTCTTAGCGCGTTGGTACA	ACACAGCATTGTGATTCTCCC	54.7	55.4
CYP3A4	Abou-Donia 2008	252	GTGGGGCTTTTATGATGGTCA	ACATCTCCATACTGGG-CAATGA	55.2	56
CYP2D6	Abou-Donia 2008	288	CCCTAAGGGAACGACAC-TCATC	ACCAGGAAAGCAAAGA-CACCA	57	57
Control Primers						
ACTB	Olivier-Van Stich-elen Lab	88	GCACTCTTCCAGCCTTCC	TGTCCACGTCACACTTCATG	55.9	55.1

Primers for human genes relevant to liver detoxification and health and control reference gene. Primers correspond with mRNA products assayed in Figure 1.

Table S2. Log files from Autodock VINA (ADV) molecular docking experiments and manually assigned pocket designation.

**Verapamil**	pocket	affinity (kcal/mol)	dist from rmsd l.b.	best mode rmsd u.b.	**Acesulfame Potassium**	pocket	affinity (kcal/mol)	dist from rmsd l.b.	best mode rmsd u.b.
1	1	-6.645	0	0	1	3	-5.186	0	0
2	1	-6.54	2.715	5.352	2	2	-4.955	17.32	18.15
3	1	-6.501	2.03	3.741	3	3	-4.95	2.516	3.185
4	1	-6.431	2.449	4.456	4	2	-4.945	16.98	17.93
5	1	-6.413	0.8102	1.804	5	3	-4.878	2.572	3.659
6	1	-6.405	1.956	2.91	6	2	-4.796	17.32	18.1
7	1	-6.38	0.9522	2.432	7	2	-4.74	16.8	17.71
8	1	-6.346	2.285	4.047	8	3	-4.716	2.589	3.468
9	1	-6.321	1.587	2.33	9	3	-4.618	16.21	17.13
10	1	-6.313	2.5	4.969	10	out	-4.613	11.88	12.6
11	1	-6.266	1.575	3.229	11	2	-4.603	8.608	9.19
12	1	-6.247	2.138	3.634	12	3	-4.532	17.12	18.08
13	1	-6.209	3.025	7.351	13	3	-4.507	17.21	18.02
14	1	-6.183	2.842	4.833	14	2	-4.503	14.59	16
15	1	-6.161	2.568	4.636	15	2	-4.5	6.524	7.379
16	1	-6.142	2.884	5.315	16	3	-4.493	7.558	8.035
17	1	-6.109	2.582	4.516	17	1	-4.483	7.686	8.661
18	1	-5.996	2.908	6.614	18	1	-4.459	6.667	7.513
19	1	-5.996	1.411	2.405	19	1	-4.39	6.899	7.731
20	1	-5.947	2.06	4.29	20	out	-4.388	6.122	6.764

**Sucralose**	pocket	affinity (kcal/mol)	dist from rmsd l.b.	best mode rmsd u.b.	**Sucrose**	pocket	affinity (kcal/mol)	dist from rmsd l.b.	best mode rmsd u.b.
1	2	-6.575	0	0	1	2	-5.723	0	0
2	1	-6.194	9.119	12.85	2	2	-5.626	1.697	4.643
3	1	-5.903	10.99	13.46	3	1	-5.534	12.46	15.15
4	1	-5.845	8.054	11.68	4	2	-5.528	1.526	3.76
5	1	-5.741	13.45	15.95	5	1	-5.482	12.4	15
6	1	-5.632	8.745	11.8	6	2	-5.413	1.702	4.12
7	3	-5.615	16.57	19.16	7	1	-5.298	9.749	12.35
8	3	-5.593	14.3	17.15	8	2	-5.298	1.642	4.139
9	1	-5.585	11.79	14.46	9	3	-5.292	17.1	19.95
10	1	-5.559	11.02	13.76	10	3	-5.278	16.99	19.28
11	1	-5.516	11.45	15.09	11	1	-5.228	11.67	14.25
12	1	-5.514	14.16	16.77	12	1	-5.185	13.47	15.45
13	1	-5.489	14.49	16.65	13	3	-5.102	15.44	17.99
14	3	-5.472	14.47	17.34	14	3	-5.048	16.01	18.68
15	1	-5.47	7.957	10.74	15	2	-5.036	2.015	3.753
16	1	-5.464	13.48	16.54	16	3	-5.016	16.01	18.34

17	1	-5.434	11.01	14.01	17	3	-5.011	17	19.69
18	1	-5.428	13.44	17.18	18	1	-4.939	12.73	15.67
19	1	-5.363	12.73	14.91	19	3	-4.912	15.42	17.73
20	1	-5.275	12.32	15.26	20	1	-4.871	13.97	16.58

Docking positions are given on the far left column for each docked compound. Pocket assignments were given following visual inspection of docking pose within transmembrane cavity on PyMOL. Affinity (kcal/mol), dist from rmsd l.b., and best mode rmsd u.b. calculated automatically by ADV.
